# Case reports: A helping hand to generalists

**DOI:** 10.1186/1752-1947-2-311

**Published:** 2008-09-26

**Authors:** Geoff Wong

**Affiliations:** 1 Walport Clinical Lecturer and GP Principal, Research Department of Primary Care and Population Health, University College London Medical School, 2nd Floor, Holborn Union Building, Archway Campus, Highgate Hill, London, N19 5LW, UK

## Abstract

Clinical decision making can be challenging for both generalists and specialists. Case reports may assist the decision making process either by providing guidance to generalists on identifying rarer conditions or a searchable database for looking up seemingly disparate symptoms. This editorial highlights the innovations being implemented by *Journal of Medical Case Reports *and *Cases Journal *in developing an educational resource to help clinicians in decision-making.

## 

If you are a generalist (in general practice/family medicine in my case) you will see a great number of patients with a wide range of symptoms. Even if you are not, throughout your career you are likely to see patients with conditions outside of your area of expertise. In many cases, their symptoms will add up to what we would recognise as a 'medical' condition, but in up to 19% they are vague, non-specific and/or contradictory and the management of patients with such undefined symptoms can pose a daunting challenge [1]. One of the big fears with such patients is that we are missing something, and dealing with undefined symptoms can be unsettling. We will all have our own ways of dealing with patients with such symptoms and one of the avenues open to us all is to use time [2]. Time may allow the symptoms to 'mature' and evolve into a more recognisable pattern, thus allowing us to clinch the diagnosis. Time also allows us to (for example) look things up and this is where I feel *Journal of Medical Case Reports *(*JMCR*) and *Cases Journal *may provide a helping hand to the generalist.

No generalist can ever expect to know everything about every condition. The research evidence clearly shows that doctors are 'good' at dealing with conditions they treat on a regular basis and potentially over-diagnose those that they have seen recently [3]. One of the key skills is in these circumstance is in knowing when and where to look things up [4].

For the rarer conditions that clinicians dread missing, many may find that *JMCR *provides a useful resource that highlights key learning points for generalists. The team at *JMCR *are working on ways for authors who publish in the journal not only to highlight that a case they report might be of interest to generalists, but also to provide key pointers on how the condition they report on might be picked up by generalists.

When clinicians are faced with a set of seemingly puzzling symptoms, then a search of the forthcoming *JMCR *and *Cases Journal *database of cases may help to provide an answer. One of the best features of this database will be that, unlike a textbook, it is updated on a very regular basis through the constant stream of submissions from around the world of cases with educational value. As such, it forms an up-to-date database that will allow clinicians access the latest information.

More importantly this database will be made up of 'real' medical cases and so unlike a textbook the history, signs, investigatory results, treatment and outcome(s) described are neither an approximation nor an 'on average' summation of a condition. In other words, learning can take place from authentic cases and not artificial 'archetypal' ones.

At present, specialists contribute the vast majority of the cases to both *JMCR *and *Cases Journal*. This likely reflects the different ethos and professional development requirements between specialist and generalists. The publication of rare or unusual medical cases might intuitively seem to be the domain and responsibility of specialist (or even sub-specialists) as such cases may describe a new condition and so help to advance our knowledge [5]. But two points are worth making here; firstly that the first clinician to see a 'rare' or novel condition may well be a generalist (something which is most likely in health care systems that have a strong and established primary care system). Secondly, we all have a responsibility to add to the medical corpus of knowledge that will further help us to care for the patients we care for.

So the next time you see a patient, ask yourself this question, "Is there anything I have learnt from this consultation that might help my fellow clinicians?" If the answer is yes, you might well have the seeds of an interesting case report and the chance to make a difference by adding to our knowledge base and improving patient care.

You can find out more on how to contribute to the journals by reading the journals' instructions for authors, at http://www.jmedicalcasereports.com/info/instructions/ and http://www.casesjournal.com/info/instructions/. To stay up to date with the case reports published in these journals, why not sign up for our regular email alerts? You can do so for *JMCR *online at http://jmedicalcasereports.com/alerts/ and for *Cases Journal *at http://casesjournal.com/alerts/. You can search *JMCR*'s growing archive of case reports online at http://www.jmedicalcasereports.com/search.

## Competing interests

GW is a Deputy Editor for the JMCR. He is paid an honorarium for this role and does not receive any commission or payments based on the volume of publications in the JMCR.
